# Predicting environmentally responsive transgenerational differential DNA methylated regions (epimutations) in the genome using a hybrid deep-machine learning approach

**DOI:** 10.1186/s12859-021-04491-z

**Published:** 2021-11-30

**Authors:** Pegah Mavaie, Lawrence Holder, Daniel Beck, Michael K. Skinner

**Affiliations:** 1 School of Electrical Engineering and Computer Science, Washington State University, Pullman, WA 99164-2752 USA; 2 Center for Reproductive Biology, School of Biological Sciences, Washington State University, Pullman, WA 99164-4236 USA

**Keywords:** Deep learning, Machine learning, Artificial intelligence, DNA methylation, Epigenetics, Transgenerational, Epimutation

## Abstract

**Background:**

Deep learning is an active bioinformatics artificial intelligence field that is useful in solving many biological problems, including predicting altered epigenetics such as DNA methylation regions. Deep learning (DL) can learn an informative representation that addresses the need for defining relevant features. However, deep learning models are computationally expensive, and they require large training datasets to achieve good classification performance.

**Results:**

One approach to addressing these challenges is to use a less complex deep learning network for feature selection and Machine Learning (ML) for classification. In the current study, we introduce a hybrid DL-ML approach that uses a deep neural network for extracting molecular features and a non-DL classifier to predict environmentally responsive transgenerational differential DNA methylated regions (DMRs), termed epimutations, based on the extracted DL-based features. Various environmental toxicant induced epigenetic transgenerational inheritance sperm epimutations were used to train the model on the rat genome DNA sequence and use the model to predict transgenerational DMRs (epimutations) across the entire genome.

**Conclusion:**

The approach was also used to predict potential DMRs in the human genome. Experimental results show that the hybrid DL-ML approach outperforms deep learning and traditional machine learning methods.

**Supplementary Information:**

The online version contains supplementary material available at 10.1186/s12859-021-04491-z.

## Introduction

Epigenetics is defined as “molecular factors and processes around DNA that regulate genome activity independent of DNA sequence, and are mitotically stable” [1]. Epigenetic changes typically involve the induction, repression or silencing of gene expression through epigenetic modifications such as DNA methylation, histone modifications, non-coding RNA (ncRNA), and chromatin structure [2, 3]. These processes are crucial to normal development and differentiation of distinct cell lineages in the adult organism [2–4]. Alterations in epigenetics promotes patterns of gene expression that can lead to adverse clinical outcomes, such as obesity, allergies, cancer, schizophrenia, or Alzheimer’s disease, to name a few [2, 5].

DNA methylation is one of the most studied epigenetic modifications of DNA, but much remains to be learned about the underlying mechanisms. DNA methylation involves the addition of a methyl group to the fifth carbon of primarily cytosine at a CpG nucleotide site [6]. This process can alter gene expression without changing the DNA sequence. Studies show that DNA methylation influences the expression of genes and regulation of proteins [7, 8]. Although the DNA sequence does not change with environmental insults, epigenetics is dramatically altered in response to the environment [2, 3]. A variety of environmental factors such as nutrition, stress, or exposure to toxicants can alter the epigenome [3].

Furthermore, epigenetic information can be transmitted between generations in the absence of direct environmental exposure through the process of epigenetic transgenerational inheritance [9]. In several studies involving exposure to toxicants, F0 generation gestating female rats were exposed during fetal gonadal development and then the subsequent F1, F2 and F3 generations evaluated [10, 11]. The transgenerational F3 generation, with no direct exposure, was found to have a large number of disease states including kidney, mammary, ovary, prostate and testis disease [12]. Analysis of the F3 generation demonstrated differential DNA methylation regions (DMRs) that had strong statistical support and were exposure specific [13, 14]. A major challenge in this area is to identify the regions in the genome that are susceptible to epigenetic modifications that are associated with disease.

The Skinner laboratory at Washington State University has produced several datasets based on the rat genome that identify the differential DNA methylated regions (DMRs) in the F3 generation after exposure of the F0 generation to one of nine toxicants: atrazine [15], dichloro-diphenyl-trichloroethane (DDT) [16], glyphosate [17], vinclozolin [18], pesticides permethrin and N,N-Diethyl-meta-toluamide (DEET) [19], dioxin [20], jet fuel [21], methoxychlor [22], and plastics bisphenol A and phthalates [23]. Atrazine and glyphosate are commonly used herbicides. DDT is an insecticide that was used extensively in the 1950s and 1960s to combat insect-borne diseases such as malaria, but has since been banned in the USA due to adverse health and environmental effects. Vinclozolin is used as both an agricultural fungicide and pesticide. Dioxin is a highly-toxic biproduct of the manufacture of chlorinated compounds, such as some herbicides, but also occurs naturally. Jet Fuel (JP-8) is a hydrocarbon mixture used commonly by the military, but has been found to be potentially toxic to the immune system, respiratory tract, and nervous system [24]. Methoxychlor is an insecticide that was intended as a replacement for DDT, but was also banned in 2003 due to adverse health effects.

The goal of this work is to use machine learning (ML) to identify regions in the genome with susceptibility to DNA methylation alterations (i.e., DMRs) due to exposure to environmental toxicants. The aforementioned laboratory analysis has identified several DMRs in the rat genome, but a ML model trained on this data can be applied to the entire genome to identify previously unknown DMR sites [25]. ML is playing an increasingly significant role in the identification of DNA regions susceptible to epigenetic alterations (i.e., epimutations), but there are still several challenges which ML does not address [26]. First, extracting the most informative features is essential for learning accurate models, but with biomedical data, this process can be labor-intensive and requires the user to have enough background knowledge about the domain to select relevant features. This is restrictive especially for high-dimensional data, where computational feature selection methods do not scale to assess the utility of the vast number of possible subsets of features. The number of genomic features can be large, and finding relevant genomic features that help to identify epigenetic sites is still a challenge. Finally, the cases of interest (e.g., disease states) are less frequent in the data compared to the non-diseased cases, which makes the case study data set imbalanced and the process of learning and extracting patterns more difficult [26, 27].

Deep learning (DL) is now one of the most active fields in machine learning and has been shown to improve prediction performance in several domains, in particular, image and speech recognition [27–30]. DL has also been successfully applied to numerous bioinformatics tasks and has discovered complex relationships in large-scale biological data [31, 32]. One of the main strengths of deep neural networks is that the raw data fed to the first layer of the network is transformed into increasingly abstract feature representations by successively combining outputs from the preceding layer to the next layer. In the end, highly complex features are produced and used to complete the learning task. Since the feature extraction depends on the structure of a network, different data representations can be extracted using different deep neural network architectures, and these aggregated features can be combined within the final prediction layer.

Despite the recent successes, DL raises several challenges. For training a DL network and finding non-linear relationships among the training data, a large number of samples are needed. To find a general and accurate classifier, DL needs to tune millions of parameters, many more than in a traditional ML method. DL can perform better only if there is a sufficient number of samples. Another challenge for DL is that it requires significant hyper-parameter tuning to find a network that can be trained to achieve the best possible performance. These parameters include the number and type of layers, the number and type of nodes in each layer, weight initialization, learning rate, batch size, loss function, number of epochs, and optimizer. Finding the best settings can take considerable time compared to other ML approaches.

Traditional machine learning methods (e.g., support vector machine, random forest, hidden Markov model, Bayesian network, Gaussian network), as well as deep learning methods, have been applied in genomics problems such as motif discovery, predicting the deleteriousness of genetic variants, cancer detection, and gene expression inference [33–36]. More specifically, these methods have been applied to several research problems related to epigenetics. One such problem is the prediction of the methylated status of a CpG site, which is a cytosine followed by a guanine in the DNA sequence. The density of CpG sites within a DNA region is highly correlated with epigenetic effects within the region. Support Vector Machines (SVMs) and decision trees have been used to compute the methylation status of a given CpG using sequence-specific features [37, 38]. Ma et al. [39] used regression and SVM to predict continuous methylation levels across tissues. Xia et al. [40] proposed a deep learning framework using a filter group normalization method to extract features and identify poly(A) signals (PASs). The outputs of the convolutional layer in this approach are grouped and normalized within each group by a subsequent filter-group normalization layer. Umarov et al. [41] developed a deep learning approach to identify promoter regions in sequences. They used convolutional layers with and without pooling in parallel to combine positional and non-positional information of CpG content in the sequence. While the use of regression is indeed more appropriate in the context of continuous methylation measurements, this approach requires extensive data collection from a source tissue. Haque et al. [25] proposed an active learning classifier that learns to classify DMR regions in the rat genome. This method identifies important examples on which to train, while reducing the overall number of examples needed which results in the need for fewer expensive samples. One of the limitations of these traditional ML approaches is the extensive use of human-engineered features. This not only incorporates human biases into the learned model, but also prevents the predictive model from discovering novel representations.

Recent DL methods for predicting DMRs have been found to outperform traditional ML approaches [5, 42]. Wang et al. [43] used a deep learning model to predict whether a CpG site was hypermethylated by using DNA patterns and topological features. The latter consists of human-engineered features taken as input by the network model. The success of these DL networks comes from their ability to learn complex features over the set of input sequences [44]. But interpreting those features is difficult, and training a generalized model typically requires a much larger set of training data compared to traditional methods.

To overcome these challenges, a hybrid learning method is proposed, which trains a DL network and extracts features from a layer in the network. The extracted features are then used to re-represent the data for input to a traditional ML method (e.g., Random Forest [45] or XGBoost [46]) which requires smaller amounts of data to achieve high accuracy. The hybrid method has the added benefit of using the DL network to visualize sequence motifs corresponding to the extracted features and using the ML method to rank the importance of these features for prediction task. The hybrid DL-ML method is particularly well-suited for DNA sequence prediction tasks, and results show that the hybrid method outperforms DL alone and ML alone for DMR epimutation prediction.

The proposed hybrid model has several advantages over other hybrid and non-hybrid approaches. First, many hybrid approaches used unsupervised learning to generate the DL features. In this work, the process of generating features is supervised, so these features are more customized to the distinguishing characteristics of DMRs. Second, by choosing XGBoost, the hybrid model can more effectively deal with imbalanced data. Third, using XGBoost to make the final prediction helps to reduce the need for hyper-parameter tuning. Finally, the hybrid model needs less data compared to DL networks.

## Results

The goal is to build a classification model that takes a region of the genome as input and predicts the region’s susceptibility to develop an environmentally induced transgenerational alteration in differential DNA methylation regions (DMRs) in the F3 generation from an ancestrally exposed F0 generation (great grandmother). The Skinner laboratory at Washington State University has produced several datasets based on the rat genome that identify DMRs in the F3 generation male sperm after exposing the F0 generation to one of nine toxicants: atrazine [15], dichloro-diphenyl-trichloroethane (DDT) [16], glyphosate [17], vinclozolin [18], pesticides [19], dioxin [20], jet fuel [21], methoxychlor [22], and plastics [23].

In these studies, the F0 generation consisted of gestating female rats that were divided into ‘control’ (no exposure) and ‘exposure’ (exposed to the toxicant) groups. The offspring of the F0 generation comprised the F1 generation. Males and females in the control or exposure groups of the F1 generation were bred to obtain the F2 generation. The F2 generation rats were bred to obtain the F3 generation. The initial direct exposure of the gestating female F0 generation rats also exposes the developing F1 generation fetus and the germ cells within the F1 generation, resulting in a direct exposure to the F2 generation. Therefore, the F3 generation represents the first descendants with no direct exposure to the toxicant. Identification of differential DNA methylated regions (DMRs) of the DNA between the control and exposure lineage F3 generations indicates that the DMR was exposure-induced through epigenetic transgenerational inheritance [3].

The procedure for identifying DMRs in the transgenerational F3 generation involved a methylated DNA immunoprecipitation (MeDIP) procedure followed by next-generation sequencing (MeDIP-Seq) [47]. The genome was divided into 1000 bp regions, and DMRs with a specific pathology were identified. A p-value was calculated for each of the 1000 bp regions indicating the probability the region is not a DMR (non-DMR). Those regions whose p-value < 10^–5^ comprise the final set of DMRs, which constitute the positive examples (DMRs) in the set of training examples used to train the hybrid model, as described in the Methods. Learning a general model with high predictive accuracy regardless of exposure is one of the major goals for this work. Therefore, when including data from multiple exposure datasets, a region is labeled as DMR if it is a DMR in any of the exposures. This model is validated by using a fivefold cross-validation test which reports the performance averaged over five trials, where each trial leaves out a different 20% of the dataset as a test set to validate the performance of the model trained on the other 80% of the dataset.

One of the main issues with epigenetic and most biological datasets is that they are naturally imbalanced, such that is, the fraction of data exhibiting the phenomenon of interest if much smaller than the alternative data. In these experiments, the number of DMRs meeting the p-value threshold is a small fraction of the entire genome. However, regions that do not meet the p-value threshold are not necessarily non-DMRs. Thus, we seek a definition of a non-DMR that makes sense biologically and ideally is close to the number of DMRs to create a balanced training set for the learning model. Three constraints were considered for defining non-DMRs: (a) a region containing no CpGs, (b) a region which is a CpG-island (CpG-density > 10%), and (c) a region whose p-value is greater than some threshold. The regions satisfying constraint (a) are clearly non-DMRs, because differential methylation is not possible without CpGs. The number of additional non-DMRs added by also including constraints (b) and (c) was typically only 1–2% of the number of no CpG non-DMRs from constraint (a). Therefore, only regions satisfying constraint (a), no CpGs, were used as negative examples (non-DMRs) in the training set. Also, CpG islands can be considered as regions with CpG-density > 20%. In some experiments, other constraints such as (a), (b), and (c) were included in the non-DMR samples but the performance is diminished.

The hybrid learning model consists of a convolutional deep neural network whose input is a 1000 bp region of the genome and whose output is a prediction of whether the region is a DMR or non-DMR. The deep network is trained on examples from the aforementioned training set. Nodes in the convolutional layer of the network represent learned features that are useful in making the final DMR/non-DMR prediction. The training data is re-expressed using these features, and this re-expressed dataset is used to train the XGBoost classifier to predict if a 1000 bp region of the genome, expressed using the DL-based features, is a DMR or not. See the Methods section for more details on the hybrid model.

### Performance of the hybrid model for predicting DMRs

The DMR prediction problem is evaluated as a two-class binary classification task. For each chromosome, and for the whole genome, the hybrid model is trained and tested using fivefold cross-validation. That is, the training set is partitioned into five equal-sized sets, and five runs of the hybrid learning procedure are conducted, where each run uses one of the five partitions as the test set and the other four partitions as the training set. The results of the five runs are averaged to yield the final results. Table 1 shows the accuracy, F1 score, precision, and recall of the hybrid model along with the number of DMR and non-DMR examples in the dataset. For each individual chromosome a separate hybrid model is trained and tested using fivefold cross validation on the DMRs and non-DMRs for that chromosome. The ALL results are for a separate model trained and tested on all the DMRs and non-DMRs across the entire genome. The four metrics are used to measure the performance of the model. Accuracy is the fraction of correctly identified DMRs and non-DMRs from the training set. Precision is the number of correctly identified DMRs divided by the number of predicted DMRs from the training set. Recall is the number of correctly identified DMRs divided by the total number of DMRs in the training set. F1 score estimates the balance between precision and recall. It is calculated as one-half times the product of precision and recall divided by the sum of precision and recall. As can be seen in the table, the hybrid model achieves high performance for these metrics.

**Table 1:** DMR prediction performance of the hybrid model using fivefold cross-validation

Chr	#nonDMRs	#DMRs	Accuracy	F1 score	Precision	Recall
1	13,959	5307	0.9643	0.9450	0.9304	0.9601
2	14,090	3990	0.9815	0.9572	0.9692	0.9456
3	7742	2664	0.9705	0.9467	0.9291	0.9653
4	7199	2900	0.9710	0.9500	0.9314	0.9695
5	7538	2805	0.9639	0.9339	0.9078	0.9616
6	6556	2151	0.9710	0.9459	0.9300	0.9623
7	6636	2349	0.9458	0.8810	0.9096	0.8818
8	4676	1955	0.9617	0.9366	0.9220	0.9517
9	5136	1867	0.9378	0.8906	0.8460	0.9403
10	2728	1804	0.9323	0.9220	0.8756	0.9737
11	3145	1365	0.9498	0.9229	0.9018	0.9451
12	2502	1284	0.9540	0.9405	0.9030	0.9812
13	5471	1789	0.9516	0.9032	0.9042	0.9022
14	5895	1844	0.9647	0.9296	0.9333	0.9260
15	4934	1802	0.9513	0.9157	0.8986	0.9337
16	4286	1500	0.9559	0.9176	0.9073	0.9281
17	3606	1533	0.9274	0.8846	0.8079	0.9777
18	3591	1425	0.9421	0.8995	0.8651	0.9368
19	2108	1195	0.9416	0.9208	0.9042	0.9382
20	1550	1023	0.9440	0.9247	0.8739	0.9810
X	13,664	1699	0.9654	0.8206	0.9096	0.7476
Y	151	79	0.8842	0.8423	0.7246	1.0
All	126,163	44,330	0.9753	0.9502	0.9556	0.9488

For each chromosome, and for ALL chromosomes, the table shows the number of training non-DMRs (#nonDMRs), the number of training DMRs (#DMRs), and the performance metrics for each model: Accuracy, F1 score, Precision and Recall

**Fig. 1:**
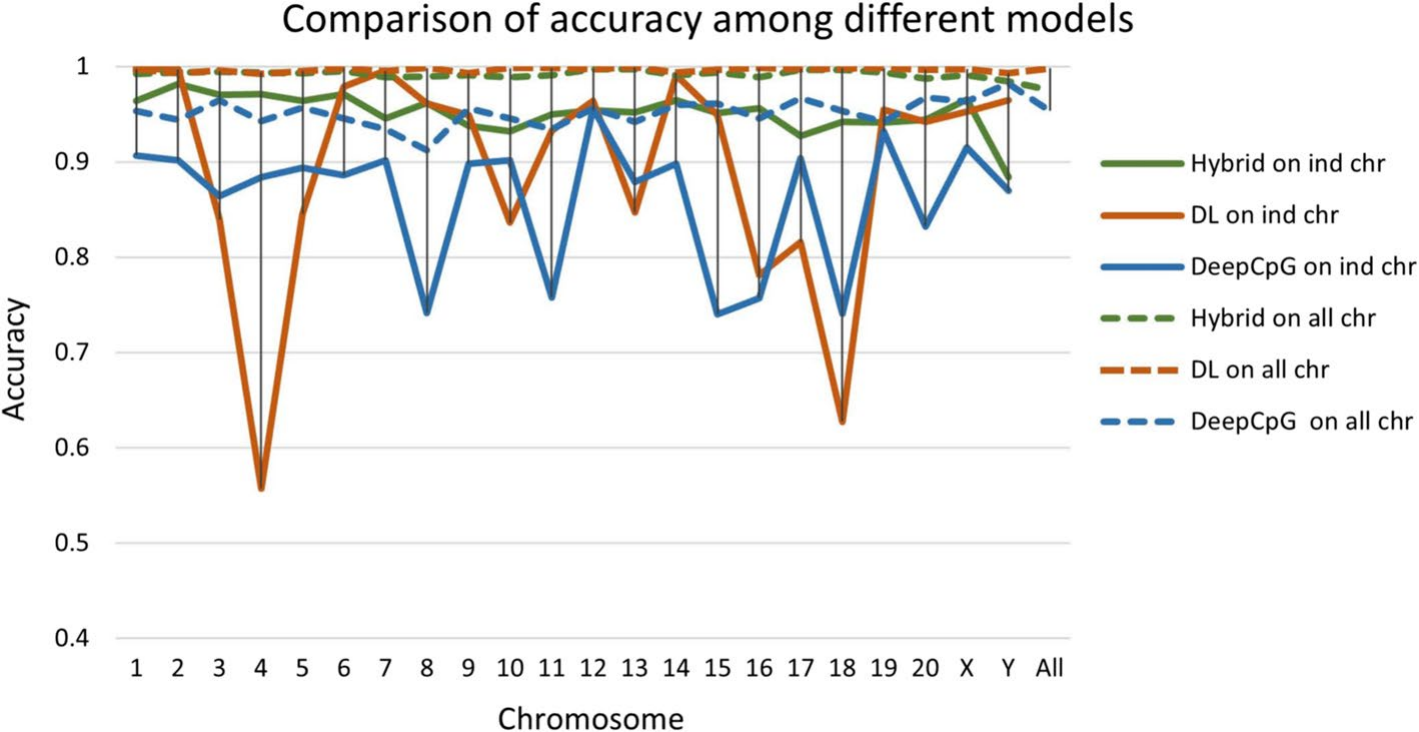
The accuracy of different models (Hybrid, DL, DeepCpG) for DMR prediction. The solid lines show the accuracy of each of the three approaches when trained and tested on the DMRs and non-DMRs for an individual chromosome ‘ind chr’. The dashed lines show the accuracy of the three approaches when trained on the DMRs and non-DMRs across the entire genome ‘all chr’. The ‘all chr’ points for the individual chromosomes are the accuracies of these ‘all chr’ models on the DMRs and non-DMRs for individual chromosomes. The points at the far right for ‘All’ are the accuracies of these ‘all chr’ models on all the DMRs and non-DMRs across the entire genome

For benchmarking purposes, the hybrid model is compared to three standalone deep learning (DL) models: DanQ, DeepSEA, and DeepCpG. DanQ [48] uses a hybrid convolutional and recurrent deep neural network for classifying the function of DNA sequences. DeepSEA [49] uses a deep convolutional model which was originally used to predict the noncoding variant effects of a sequence. DeepCpG [50] uses multiple DL network modules to predict the presence of methylated CpGs in a DNA sequence. For comparison to the hybrid approach DeepCpG is modified to produce a binary classification (DMR or non-DMR) for the sequence. DeepCpG is a deep learning approach that utilizes a convolutional network to model the DNA sequence and a fully connected network to model the neighborhood of CpGs. There are several options which can be used to refine these networks. In the modified DeepCpG model a dense layer with two nodes is added to perform the final binary classification. To compare performance among these models, the accuracy, F1 score, precision, and recall for each model is calculated. For models trained on individual chromosomes, the hybrid model outperforms the rest. The hybrid model was tested on 22 chromosomes, and the average of the accuracy of the hybrid model on each chromosome individually is 95.14%; whereas, the average accuracy of the standalone DL model is 89.45%, for DanQ is 93.32%, for DeepSEA is 92.53%, and for DeepCpG is 86.19%. Figure 1 and Additional file 2: Fig S2 show the performance of the models based on their accuracy scores. In Fig. 1 the solid lines show the accuracy of each of the three approaches when trained and tested on the DMRs and non-DMRs for an individual chromosome ‘ind chr’. The dashed lines show the accuracy of the three approaches when trained on the DMRs and non-DMRs across the entire genome ‘all chr’. The points for the individual chromosomes are the accuracies of these all chromosome models on the DMRs and non-DMRs for individual chromosomes. The points at the far right for ‘All’ in Fig. 1 are the accuracies of these all-chromosome models on all the DMRs and non-DMRs across the entire genome.

The results in Fig. 1 and Fig S2 show that the hybrid model outperforms the other models in most cases, but there are some exceptions. In chromosome X, the training dataset is imbalanced, and the number of DMRs in the training samples is low, which is likely the reason for the low recall performance of the model. The model is more biased toward predicting a region as a non-DMR. In chromosome Y, recall is 1.0, which means that all the errors are false positives (i.e., incorrectly predicting DMR). One possible explanation is that the Y chromosome has a large number of repeat elements, which means higher variability is anticipated. In addition, the number of X chromosome training samples is low, which is a challenge for any machine learning method.

Figure 1 also shows that the three models (Hybrid, DL-alone, DeepCpG), when trained on all the data from entire genome, outperform the models trained for individual chromosomes. The main explanation for this increased performance is the general result that deep learning methods perform best when large numbers of training examples are available. In this scenario the performance of the DL-alone model rivals that of the Hybrid model. However, as discussed below, the DL-alone model is significantly more general in that it predicts over 600 K additional regions in the genome to be DMRs. Additional testing has shown that the Hybrid model is better at incorporating other handcrafted features. When these features are added to the inputs for the DL network, the resulting model performs worse than when these features are added at the non-deep learning phase of the model (i.e., as additional inputs to the XGBoost classifier). Therefore, use of a DL-alone model is warranted only when large amounts of training data are available.

The prediction performance of the rat model on the rat genome was validated using a five-fold cross-validation experiment, which is a well-known statistical test used to evaluate machine learning methods. For each fold, the experiment trains a model on 80% of the data and uses the remaining 20% as a test set. This technique is repeated five times, and each time the model is tested on a different 20% of the data after being trained on the remaining 80%. The final performance is the average over the five folds. The cross-validation performance is considered an unbiased estimate of the performance of the model trained on the entire dataset. The performance values in Table 1 show the results of this five-fold cross-validation experiment.

### Whole-genome epimutation prediction

The hybrid model is further evaluated by using it to classify each region across the whole genome as to whether or not the region is susceptible to form a DMR in response to an ancestral environmental induced exposure. In this experiment, the hybrid model is trained on the entire dataset, not a fivefold method as in the previous section. Table 2 shows the number of predicted DMRs in each chromosome and the whole “ALL” genome (#Predicted DMR column), the percentage of the entire chromosome/genome predicted to be DMRs (%Genome column), and the percentage of the training DMRs correctly predicted (%Recall column). As a comparison to the number of predicted DMRs in the whole genome, an upper bound on this number would be the number of regions with at least one CpGs, i.e., the complement of the non-DMR set. This complement of the non-DMR set is called “maximum possible DMRs”. This maximum possible DMRs set contains all the regions in the genome except those in the non-DMR training set. Table 2 also shows the number of maximum possible DMRs in the genome and the percentage of the genome that contains these maximum possible DMRs. While the percentage of the genome that the hybrid model classifies as a DMR is high, it is still well below the upper bound. These predicted DMRs that are not in the original training set represent areas of the genome that warrant further study for susceptibility to become transgenerational DMR from ancestral environmentally induced exposures.

**Table 2:** DMR prediction performance of hybrid model learned from all training data

Chr	#Predicted DMR	%Recall	#max poss. DMRs	%max poss. DMRs	%Genome
1	127,816	96.09	267,040	95.03	45.54
2	141,619	85.88	250,909	94.68	53.44
3	105,790	92.90	168,257	94.52	60.10
4	159,974	97.34	152,775	95.49	87.41
5	74,363	94.15	164,461	95.61	43.23
6	103,072	93.77	139,443	95.50	70.59
7	76,805	83.48	137,363	95.39	53.33
8	57,713	97.74	127,323	96.45	43.72
9	73,307	96.03	115,863	95.75	60.58
10	93,641	87.19	108,271	97.54	84.36
11	39,174	87.54	85,854	96.46	44.01
12	43,108	91.82	110,497	97.78	84.52
13	44,317	96.31	108,528	95.00	39.21
14	62,311	94.63	104,104	94.64	54.65
15	96,840	74.41	84,065	94.45	88.03
16	62,548	94.86	84,713	95.18	70.27
17	71,651	98.56	83,393	95.85	80.50
18	53,671	98.38	83,408	95.87	61.69
19	51,335	88.20	58,891	96.54	84.18
20	18,205	98.33	47,449	96.83	37.12
X	47,092	68.45	144,335	91.35	2.98
Y	2608	91.13	3159	95.43	85.94
ALL	1,748,888	95.49	2,742,978	95.40	63.75

The number of DMRs in a chromosome predicted by the hybrid model trained on data from that chromosome, and the number of DMRs predicted across the whole genome (ALL) by the hybrid model trained on data from the whole genome (#Predicted DMR). Also shown is the percentage recall (%Recall), which is the percentage of the training DMRs that the model correctly predicts as DMRs. As a comparison, “maximum possible DMRs” is defined as the set of all 1000 bp regions minus those regions that are clearly nonDMRs, because they have no CpGs or more than 20% (200) CpGs. The size of this “maximum possible DMRs” set serves as an upperbound on the number of possible DMRs, and the number of predicted DMRs should be well below this bound. The table shows the size of this set (# max poss. DMRs) and the percentage of the chromosome or whole genome this set represents (% max poss. DMRs). The %Genome column shows the percent of the chromosome, or entire genome for ALL, that the predicted DMRs represent. The %Genome value should be well below the “% max poss. DMRs” value

**Fig. 2:**
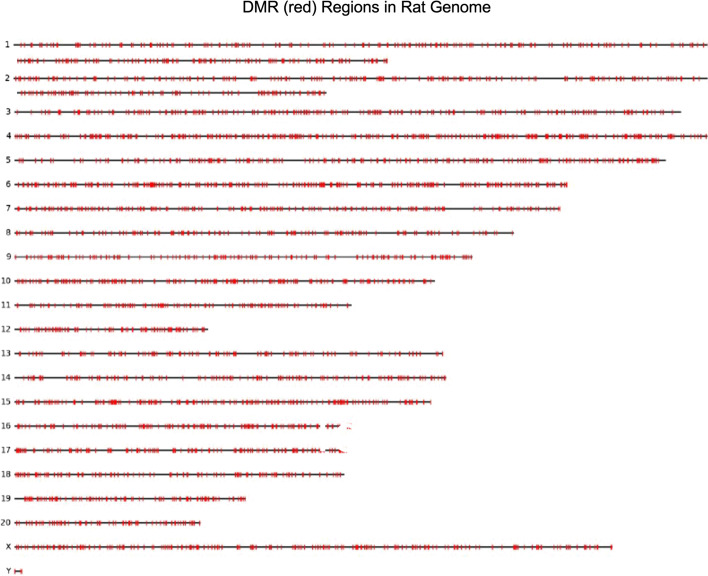
Visualization of the chromosomal locations of the predicted DMRs in the rat genome, where 0.02% of the predicted DMRs are randomly sampled from Table 2 and depicted in red. The 0.02% sampling rate was selected to represent the distribution of DMRs across the genome while still being able to visualize variations in density. The predicted DMRs are distributed equally across the whole genome

Figure 2 shows a visualization of where these predicted DMRs reside in the rat genome. For visualizing the distribution of DMRs in the whole genome, 0.02% of the predicted DMRs are randomly sampled from Table 2 and depicted in red in Fig. 2. The 0.02% sampling rate was selected to represent the distribution of DMRs across the genome while still being able to visualize variations in density. The figure shows the predicted DMRs are generally distributed equally across the whole genome. However, there are some regions in each chromosome with high DMR density.

**Fig. 3:**
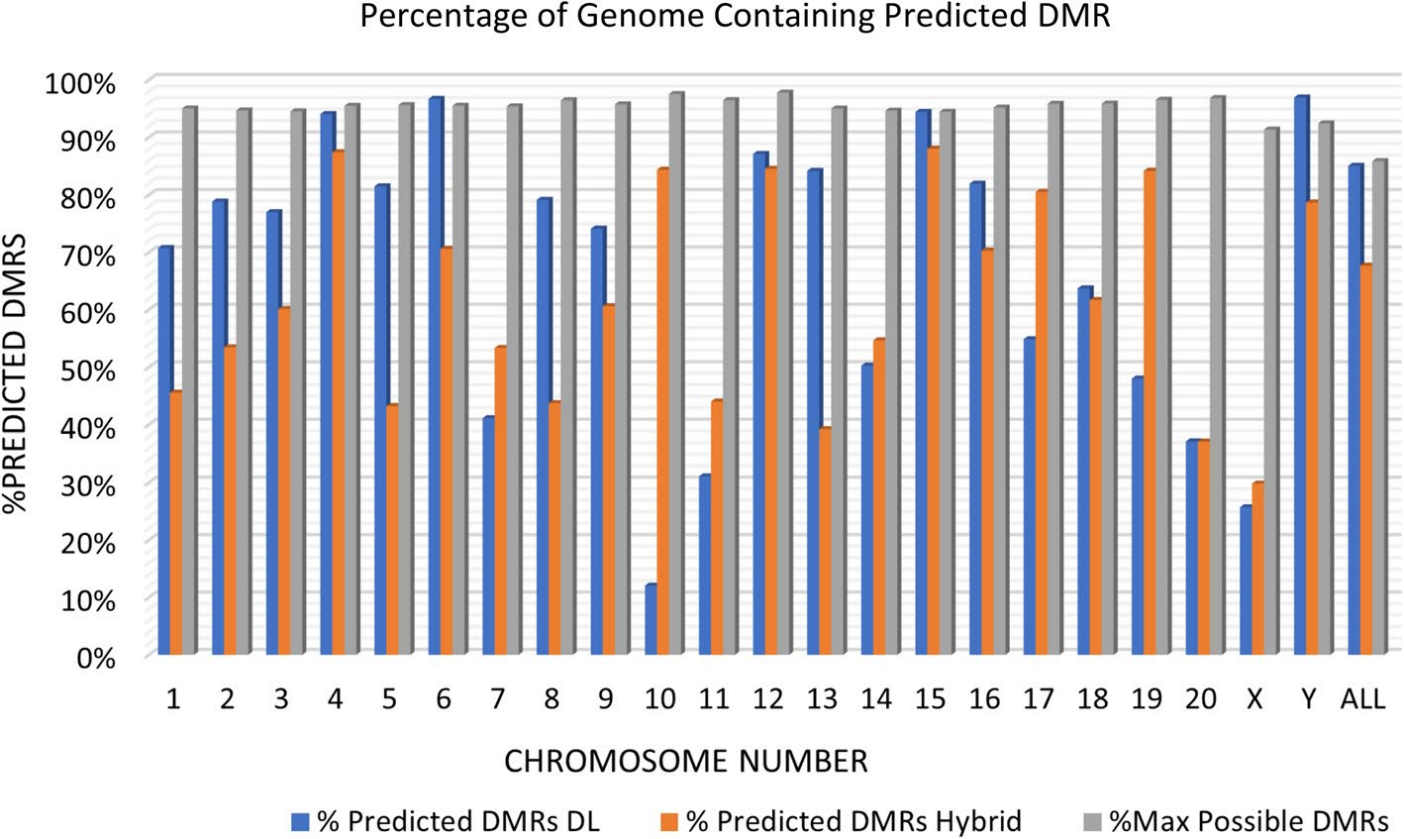
A comparison between the percentage of predicted DMRs in the genome using different models. The “% DMRs DL” is the percentage of the chromosome (or entire genome for ALL) where the DL model predicts DMRs. The “% DMR Hybrid” is the percentage of the chromosome (or entire genome for ALL) where the hybrid model predicts DMRs. The “% Max Predicted DMRs” is the same as the “% max poss. DMRs” in Table 2 (see definition there), which represents an upper bound on the percentage of the chromosome (or entire genome for ALL) that are possible DMRs

These results show a trade-off between two objectives for training the hybrid model, i.e., maintaining high model accuracy while avoiding overly general predictive models. Table 1 and Fig. 1 show that the hybrid model achieves high accuracy compared to alternative approaches. Table 2 and Fig. 2 show that while the number of predicted DMRs represents a significant percentage of the genome, the number is still well below the number of possible DMRs. To further illustrate this trade-off, Fig. 3 shows the percentage of the genome represented by the predicted DMRs using the Hybrid model and the standalone deep learning (DL) model. The figure also shows the percentage of the genome represented by the upper bound on the possible DMRs. The results show that the Hybrid model predicts far fewer DMRs than the upper bound, and in most cases, fewer than the standalone DL model. Therefore, the hybrid model effectively trades off the objectives of a high accuracy, but a not-overly-general model.

**Fig. 4:**
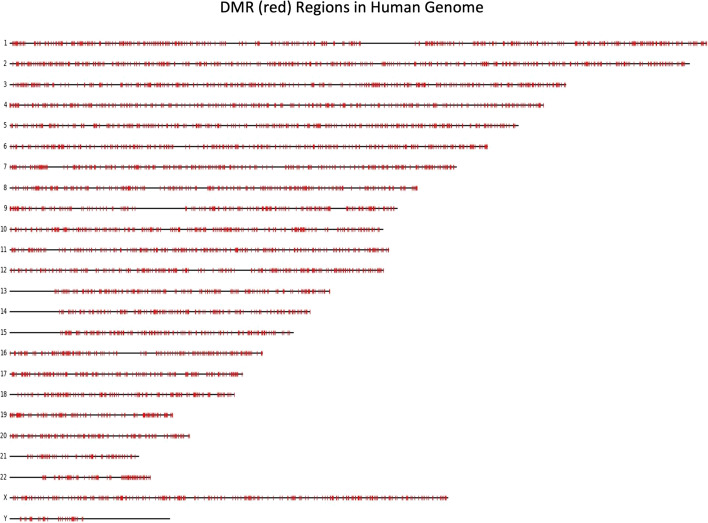
Visualization of the locations of the DMRs in the human genome, as predicted by the rat model, where 0.02% of the predicted DMRs are randomly sampled from all predicted DMRs and depicted in red. The 0.02% sampling rate was selected to represent the distribution of DMRs across the genome while still being able to visualize variations in density. Predicted DMRs are distributed equally across the genome with some regions having a higher DMR density than others

The rat model can also be applied to the human genome in order to identify potential conserved DMRs between the two organisms. Figure 4 shows a visualization of the locations of the DMRs in the human genome, as predicted by the rat model, where 0.02% of the predicted DMRs are randomly sampled from all predicted DMRs and depicted in red. The 0.02% sampling rate was selected to represent the distribution of DMRs across the genome while still being able to visualize variations in density. Similar to the predicted DMRs in the rat genome shown in Fig. 2, Figure 4 shows the predicted DMRs are generally distributed equally across the human genome with some regions having a higher DMR density than others. However, there are significant gaps in several chromosomes, where the sequence is mostly repeat elements or N’s and so the model predicts non-DMR in those regions. The hybrid model predicted 1.748 × 10^6^ potential DMR sites in the rat genome (Table 2), and 2.19 × 10^6^ potential DMR sites in the human genome.

### Deep network feature visualization

**Fig. 5:**
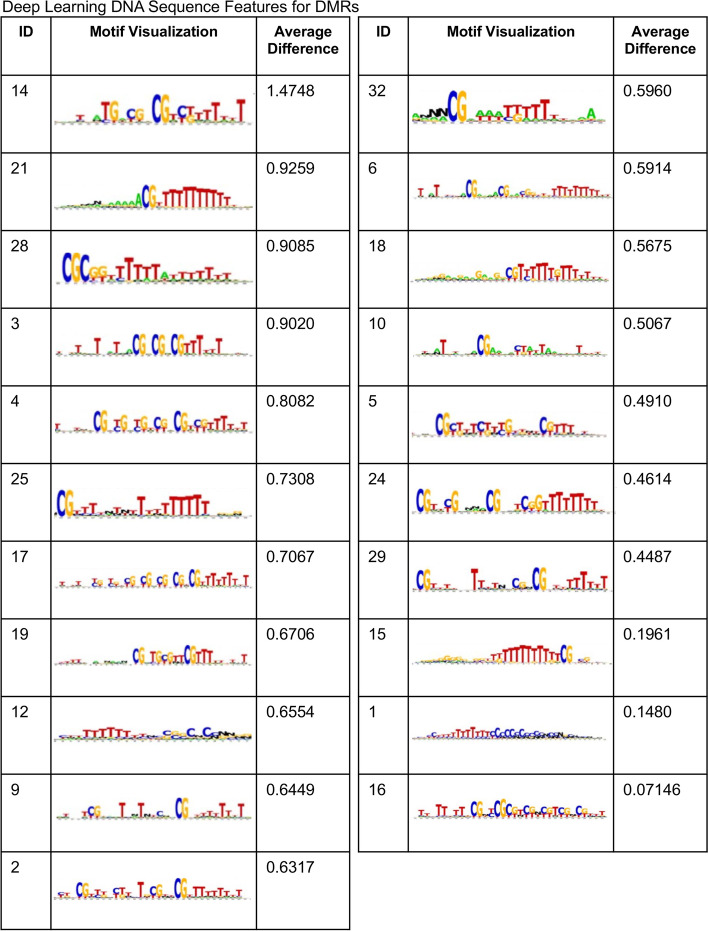
Deep learning DNA sequence features for DMRs. Sequence motif visualizations for the 21 DMR detector features out of the 32 features extracted from the DL model. DMR detectors are those features whose average activation for DMR examples is greater than for non-DMR examples. The feature ID, motif visualization, and the difference between the average DMR activation and the average non-DMR activation are presented. A larger difference indicates a feature motif more biased toward DMRs

The previous results indicate that the features extracted from the DL network, and used to train the XGBoost ML classifier, are effective for learning to classify a region as a susceptible transgenerational DMR from ancestral environmental exposures. The ability to interpret these features and determine their biological relevance is important to further validate the approach and understand the properties of a DMR region. One capability with using a DL network is the ability to visualize what properties of the input sequence trigger each of the extracted features. In particular, for each kernel in the convolutional layer of the DL network corresponding to an extracted feature, the distribution over the possible base pairs (A, G, C, T, N) at each location of the 1000 bp input sequence that causes this kernel’s activation to exceed some threshold can be computed over all the training examples. Furthermore, the features can be ranked based on their utility in XGBoost for classifying the examples and categorized as more instrumental in classifying a region as a DMR or as a non-DMR. Figure 5 and Additional file 1: Fig S1 show the kernel motif visualizations using the Deepomics tool and pysster [51, 52]. For each feature, the average activation of the feature is computed for the DMR training examples and for the non-DMR training examples. The feature can be said to focus on the class leading to the larger average activation. These motifs are divided based on the average weights for positive (DMR) and negative (non-DMR) classes. If the average output of a kernel for the positive examples is higher than the average output for negative examples, it is considered as a DMR detector kernel. In contrast, if lower, it is considered as a non-DMR detector. The motifs in Fig. 5 and Additional file 1: Fig S1 are sorted based on the difference between the average output for the positive and negative examples. Figure 5 shows the motif visualization for kernel features that are DMR detectors, and Additional file 1: Fig S1 shows the motif visualization for kernel features that are non-DMR detectors. The motif visualizations show frequent occurrences of CpGs (80 in all), which is an important factor for a DMR. CpGs are shown frequently in both DMR and non-DMR detector motifs, but these motifs depict important features for the decision process. The occurrence of CpGs in non-DMR detectors can be seen as a factor for deciding DMRs or non-DMRs, because if the region has no CpGs, then it would indicate this region is a non-DMR.

**Fig. 6:**
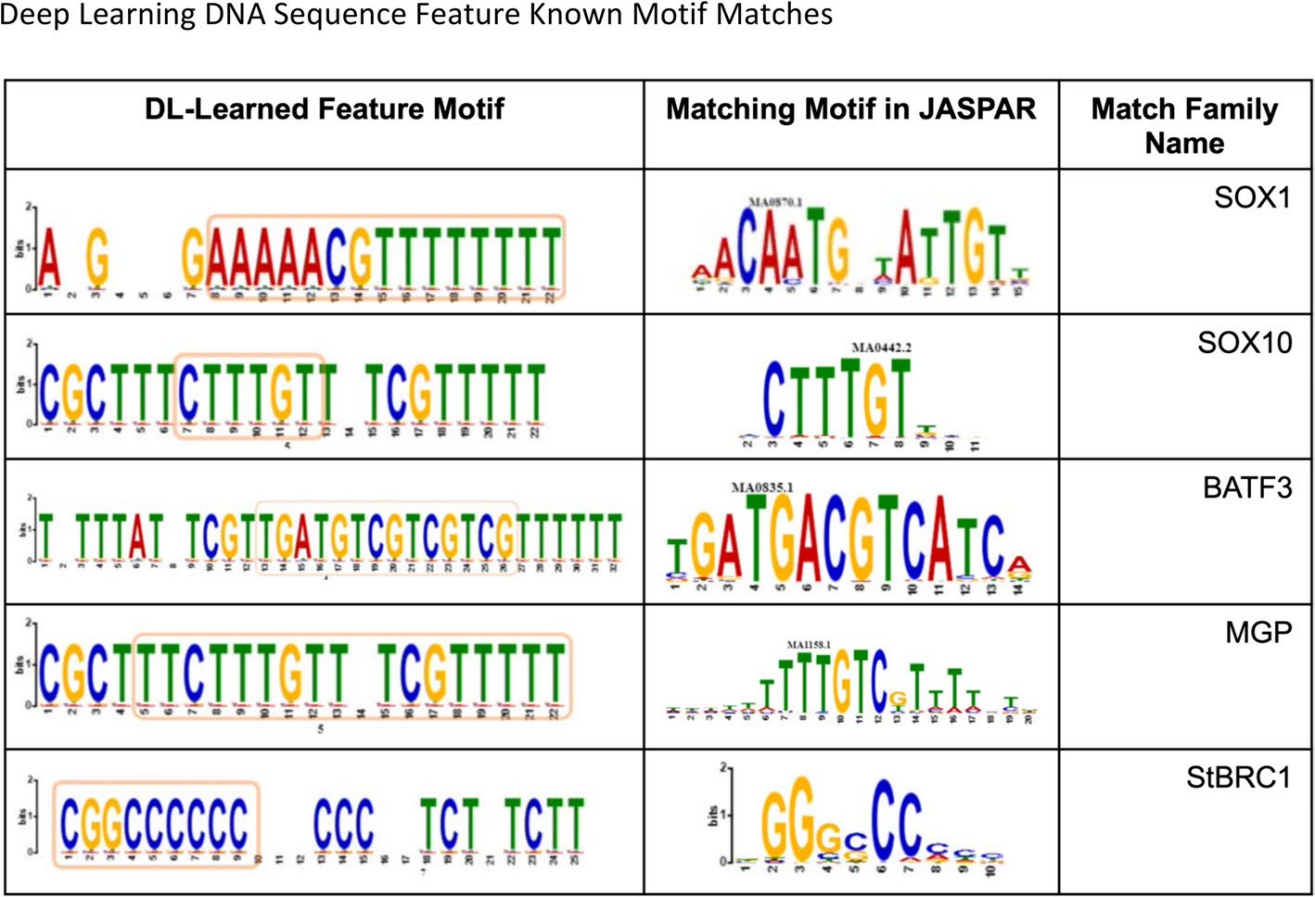
Deep Learning DNA Sequence Feature Known Motif Matches. The 32 DL-learned features from Figs. 6 and S1 were converted to DNA sequence motifs (highest-weighted base-pair in each location) and input to the Tomtom motif matching tool against the JASPAR transcription factor motif database. Five of the matches, including the DL-Learned Feature Motif, the matching motif in JASPAR, and the family associated with the matching motif are presented

To perform a more systematic evaluation of each feature’s biological significance, the Tomtom tool [53] is used to align the DL feature motifs with known motifs. For the 32 feature motifs learned by the hybrid model, Tomtom found 187 matches to known motifs. Figure 6 shows an example of five of the hybrid model’s features along with their matching motifs. Among the matching motifs are 26 from the SOX (SRY-related HMG-box) family of motifs such as SOX1, 2, 6, 3, 10, 13, and 15. The importance of SOX10 (NCBI Gene ID 6663) in the susceptibility of a region has been noted in previous work [54]. The extracted motifs also matched with SOX1 (NCBI Gene ID 6656) using JASPAR (non-redundant) as the reference dataset. The NCBI summary FOR SOX1 states “This intronless gene encodes a member of the SOX family of transcription factors involved in the regulation of embryonic development and in the determination of the cell fate. The encoded protein may act as a transcriptional activator after forming a protein complex with other proteins. In mice, a similar protein regulates the gamma-crystallin genes and is essential for lens development.” Another known motif highly matched with the kernel visualization motifs is SOX10. The NCBI summary for SOX10 states “This gene encodes a member of the SOX (SRY-related HMG-box) family of transcription factors involved in the regulation of embryonic development and in the determination of the cell fate. The encoded protein may act as a transcriptional activator after forming a protein complex with other proteins. This protein acts as a nucleocytoplasmic shuttle protein and is important for neural crest and peripheral nervous system development. Mutations in this gene are associated with Waardenburg-Shah and Waardenburg-Hirschsprung disease.”

## Discussion

Epigenetic effects of exposures through DNA methylation are strongly related to disease development [3]. In addition to the identification of DMRs in the direct exposed F0 and F1 generations, the great grand-offspring F3 generation can also be analyzed for the presence of disease (e.g., testis, prostate and kidney disease, obesity, polycystic ovaries, reduced oocyte number in the ovaries, and cancer) [10] and have correlation to specific diseases to specific exposures. Predicting regions of the genome susceptible to develop into transgenerational epimutations and understanding the important features of a region for making the prediction will improve the ability to diagnose and prevent these diseases [26]. This paper proposes a hybrid model that predicts a DNA region’s likelihood to be differentially methylated (DMR) as a result of ancestral exposure to environmental toxins. The hybrid model is composed of two components, a deep learning network for learning new features and a non-deep learning machine learning classifier, that enable the model to provide more accurate predictions than either component alone, as well as extract meaningful features.

The hybrid approach has been used in machine learning to improve the performance of prediction tasks [55]. Previous studies indicate that combining several techniques shows better performance than single techniques [56]. There are several types of combinations for developing hybrid models. As an example, a hybrid model can consist of one unsupervised learner for preprocessing (extracting features) and one supervised learner as a classifier. A hybrid model can contain supervised or unsupervised components. Yang et al. [57] developed a hybrid tool for electricity price forecasting by combining the kernel extreme learning machine (KELM) with an autoregressive moving average (ARMA) (two traditional machine learning algorithms). Choudhry and Garg [58] proposed a hybrid machine learning system based on a genetic algorithm and support vector machine for stock market prediction.

As in this work, the hybrid approach can improve performance by combining deep learning and traditional machine learning methods. Tsai et al. [59] used a hybrid neural network and decision tree model for stock price forecasting. Wan et al. [60] proposed Neural Backed Decision Trees, altered hierarchical classifiers that use trees built in weight space. Their model is accurate and interpretable. Kong et al. [61] introduced a classifier called Forrest Deep Neural Network which combines a deep neural network architecture with a supervised forest feature detector for learning sparse feature representations for gene expression. Kontschieder [62] proposed a stochastic and differentiable decision tree model combined with a deep learning model that the decision forest makes the prediction. Grover et al. [63] focused on combining discriminative techniques with a deep neural network to model the joint statistics of a set of weather-related variables. For predicting and evaluating the critical performance of the plasma steam reforming of tar, Wang et al. [64] developed a model that contains both an artificial neural network and a support vector machine. These hybrid approaches combine the DL and ML methods by using them in parallel and then combining their outputs or using then sequentially by feeding the output of one as the input to the other. The hybrid approach used here is novel in that it extracts knowledge learned from the DL model, in the form of new features, and uses those features to improve the performance of the ML model.

In comparison to other ML approaches for epigenetics, the proposed hybrid model does not require measuring methylation levels in the sample of interest, is not limited to specific CpGs, uses neural networks as feature extractors instead of human-engineered features, and for the classification task uses a traditional machine learning approach that typically requires fewer samples for training. While the focus here is on epigenetic datasets to predict the transgenerational DMRs, the hybrid approach can be applied to any classification task based on a genomic dataset where the prediction task is to extract interpretable features, incorporate them into a single model, and review their importance in the prediction task. Human-engineered features are not needed, because features are derived from the DNA sequence by using a Convolutional Neural Network (CNN) as a motif detector.

For training the hybrid model the raw DNA sequence is the only input to the DL network. The XGBoost classifier relies on the sequence based features constructed by the DL network to train a high performance model for DMR prediction. There is a possibility that adding other biological features may improve the performance of the model. For example, CpG density has been found to be highly correlated with DMR regions. Such features could be combined with the DL extracted features for input to the XGBoost classifier. Further evaluation is necessary to determine if the addition of such features improves the performance of the hybrid approach over using purely sequence based features.

Another alternative model would be to use only a traditional, non-deep learning classifier, such as the XGBoost classifier used in the hybrid approach. The main challenge with such approaches is the need to design and compute features from the DNA sequence to be classified. Such an approach has been taken in previous work. Haque et al. [25] utilized a combined tree-augmented naïve Bayes (TAN) classifier combined with the AdaBoost boosting method to perform DMR classification. The datasets used by Haque et al. were generated using a different method (MeDIP-chip rather than MeDIP-seq), but did originate from the same animals. The TAN + AdaBoost approach achieved comparable performance (97%) on a regenerated subset of the datasets used here, but relied on over 900 manually-chosen features derived from the DNA region. The performance of RandomForest and XGboost were evaluated on the regenerated subset of the datasets. RandomForest also achieved 96% accuracy score. The average accuracy of XGBoost was also 97%. The hybrid model achieves similar performance without the need for handcrafted features.

The model accuracy results were validated using a fivefold cross validation test that divides the data (DMRs and non-DMRs) into five non-overlapping partitions. In this test, five trials are performed where each trial sets aside one partition as the test set and trains a model on the other four partitions as the training set. The learned model is evaluated on the set aside test set. The final accuracy is the average of the of these five models on their corresponding test set partition. Cross validation is a standard approach to model evaluation in the machine learning community [65, 66].

Results show that the hybrid model has high accuracy on the data constructed from nine different exposures; however, for the regions not explicitly identified as DMR or non-DMR in the dataset, the model predicts a large fraction of these regions to be DMRs. While the true classification of these regions is not known, it is likely that the fraction of actual DMRs is lower. This leads to several hypotheses. First, the model may need further specialization. One approach to specializing the model is to identify additional negative examples (non-DMRs) to include in the training set beyond the current set of regions with no CpGs. Two other candidate sets of non-DMRs are regions with more than 20% CpGs (CpG-islands) and regions whose p-value (probability of non-DMR) is sufficiently high. Another approach to specializing the model is to use an ensemble of models. Ensemble learning is known as a class of strategies in which instead of learning a single model, there are several models involved in the decision process. There are three main approaches for ensemble learning: bagging, boosting, and stacking-based methods [67]. In the ensemble approach a region is predicted to be a DMR only if a significant fraction of the models in the ensemble predict DMR. The high variance in deep learning models is a known problem, and studies show that combining the output of several models can achieve better performance than an individual model. For example, an LSTM/CNN was used to predict the pathogenic potential of DNA sequences [68]. Zhang et al. [69] used an ensemble deep learning method to predict DNA binding sites in the protein sequences. Zacharaki et al. [70] developed a deep convolutional neural network ensemble framework for predicting protein functions. DeepCpG is another example of an ensemble approach for predicting a single base pair DNA methylation state [42].

Another approach to specializing the model is to consider exposure-specific models. Many of the DMRs for each exposure are unique, and learning a model to predict DMRs across all exposures can result in over-generalization. Therefore, learning individual models for each exposure may yield better results. For example, training a model using DMRs detected only from exposure to atrazine may be better at predicting atrazine DMRs than a model trained on DMRs from all nine exposures. That there exist several DMRs unique to one exposure (e.g., 3258 of the 7553 DMRs predicted by the model for atrazine are unique to atrazine compared to the other eight exposures) further supports the hypothesis that exposure-specific models may outperform models trained on DMRs from all exposures. Finally, the mechanism by which epigenetic effects are realized may involve a preponderance of DMRs rather than a specific DMR signature, which would lead to an over-general model if focused on finding such an elusive signature.

The ability of a DL network to construct its own features for representing and classifying regions of the genome is a powerful capability that will benefit a large set of sequence-based classification tasks. The ability to visualize these features as abstract motifs has shown that the network-derived features are more complex than typical human-derived features, but still biologically meaningful. One weakness of a pure DL approach to sequence-based classification problems is the difficulty in understanding how the network arrived at a particular prediction. Using the network-derived features to learn a traditional ML classifier allows a more detailed understanding of the prediction. In particular, decision-tree based methods like XGBoost allow a ranking of the feature importance and reveal how each feature is used to filter a test region through the decision tree to a final prediction. Visualizing not only the features, but how they are used within the non-DL classifier, will yield further insights into the underlying epigenetic mechanisms.

There are several additional directions to explore to further improve the model. First, a more systematic exploration of the DL network hyper-parameters can be performed by recently-developed hyper-parameter tuning algorithms [71]. Second, combining biological features known to correlate with DMRs (e.g., CpG-density) along with the DL-generated features to represent the data for input to the non-DL classifier may further improve performance. Third, the inclusion of additional general genomic features (e.g., structural and evolutionary properties of the DNA region) as additional inputs to the DL network has shown promise [72]. Finally, as more datasets for the rat and human genomes emerge, they can be included in the training data to improve the models.

## Conclusion

The ability to accurately predict the location of DMRs resulting from environmentally induced epigenetic transgenerational inheritance will improve the ability to identify epigenetic biomarkers for specific exposures and the exposure-specific diseases. Models trained on DMRs from specific exposures can predict the presence of DNA regions in the genome that indicate susceptibility to epigenetic mutations caused by the exposure. Models trained on DMRs from specific diseases can likewise predict the presence of DNA regions in the genome that indicate susceptibility to the disease. Since there are unique DMRs associated with each exposure, this suggests a diagnostic tool that can identify likely exposures in an individual’s ancestry and contributing causes to the presence of disease. Recently, observations indicate there are unique DMRs associated with specific diseases [17, 73, 74], so the same process can be used in a diagnostic tool for disease susceptibility. The hybrid machine learning approach proposed here can learn models that predict DMRs with high accuracy and support the implementation of diagnostic tools for exposure and disease diagnostics.

## Methods

The overall method consists of five steps: (1) train a DL model for the classification task and extract the initial convolutional layer of the DL model as features; (2) express the data using the extracted features; 3) train a traditional ML classifier on the data expressed using these features; 4) identify the most important features used for classification; and 5) visualize these features as DNA sequence motifs. The method is implemented in TensorFlow, Keras, and Scikit Learn and is available online at github.com/skinnerlab/DL-ML-Hybrid, and skinner.wsu.edu/genomic-data-and-r-code-files/, and github.com/holderlb/DL-ML-Hybrid.

**Fig. 7:**
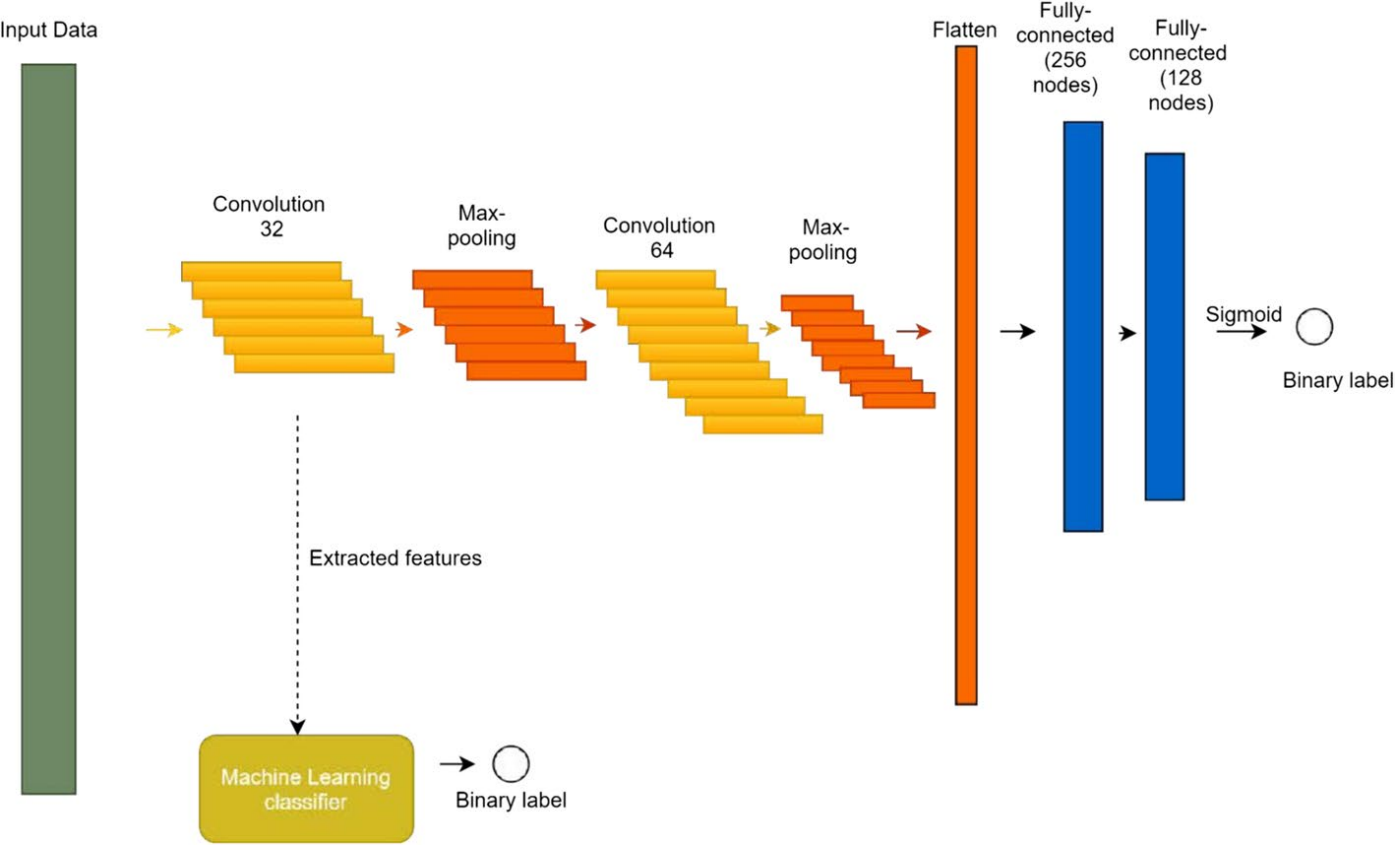
Simplified diagram of the hybrid model. A 1000 bp input DNA sequence is one-hot encoded using a 5 × 1000 binary matrix. A convolution layer transforms the input matrix into an output matrix, where each output represents a sequence motif. After each convolutional layer is a batch-normalization layer following by a ReLU transformer layer. The max-pooling down-samples the output matrix. This block is followed by another similar block consisting of a convolutional layer, following by batch-normalization, ReLU, and a max-pooling layer. The classification block begins by flattening the output matrix of the previous layer, followed by two fully connected dense layers with 256 and 128 nodes, and the last layer consists of two nodes, one for each label: DMR and non-DMR. After training the network, the output of the first convolution layer represents new features used to re-express the input sequence. The re-expressed training data can then be used as input to a traditional ML classifier

The method takes a 1000 bp region of the DNA sequence as input and produces a classification for the region as to whether it will be susceptible to environmental exposure as evidenced by differential methylation. Additional file 4: Table S2 shows a summary of the training datasets. The proposed hybrid model shown in Fig. 7 consists of a deep learning (DL) network that is trained using the dataset and a traditional machine learning (ML) classifier that is also trained using the dataset, but with the input region re-expressed using features extracted from a layer of the deep learning network.

The overall method consists of five steps: (1) train a DL model for the classification task and extract the initial convolutional layer of the DL model as features; (2) express the data using the extracted features; (3) train a traditional ML classifier on the data expressed using these features; (4) identify the most important features used for classification; and (5) visualize these features as DNA sequence motifs. The method is implemented in TensorFlow, Keras, and Scikit Learn and is available online at github.com/skinnerlab/DL-ML-Hybrid, and skinner.wsu.edu/genomic-data-and-r-code-files/, and github.com/holderlb/DL-ML-Hybrid.

The method takes a 1000 bp region of the DNA sequence as input and produces a classification for the region as to whether it will be susceptible to environmental exposure as evidenced by differential methylation. The proposed hybrid model shown in Fig. 7 consists of a deep learning (DL) network that is trained using the dataset and a traditional machine learning (ML) classifier that is also trained using the dataset, but with the input region re-expressed using features extracted from a layer of the deep learning network.

### Deep learning network

The DL network consists of a 5 × 1000 one-hot input matrix, where each row represents one of A, C, G, T, or N, with a 1 in the corresponding row and a 0 in the other four rows. This input matrix is fed into a sequence of two blocks, each consisting of a convolutional layer followed by a max-pooling layer. These layers serve to generate new complex features based on the input sequence, reduce the dimension of the previous convolutional layer for input into the next convolutional layer, and control overfitting during the learning process. Such a convolutional neural network (CNN) allows features to be constructed independently of their position in the input sequence. For each convolutional layer, we need to decide the number of filters and the kernel size. For the first convolutional block, 32 filters are used, and the size of each filter is 20. For the second convolutional block, 64 filters are used with the same size. In each block, there are two convolutional layers. The first convolutional layer does not use padding, but the second one uses padding to conserve the size of the output. A batch-normalization layer is used after each convolutional layer. This helps each layer of the network to learn more independently of other layers. Since batch normalization regulates the value of activation, the learning rate can be safely increased to accelerate the learning process, reduce overfitting, avoid activation function saturation and gradient vanishing, and increase the stability of the network. The batch-normalization layer is following by ReLU activation in each convolutional layer.

After two convolutional layers, a max-pooling layer is used to generalize the model; the pooling size for the max-pooling layer is 2. At the end of each block, a dropout layer randomly drops neurons from the network and further helps the network to overcome the overfitting problem by reducing the number of parameters. The dropout rate is 0.4.

After the convolution-max-pooling blocks is the classifier block, which contains two dense layers and a classifier layer. The first dense layer contains 256 nodes, and the second dense layer contains 128 nodes. The dense layers are responsible for combining the extracted and aggregated features and modify the feature weights using error back-propagation based on correlation to the final classification. The classifier layer is a dense layer with an output node for the label. Softmax is used for the activation function, and the output of the last layer is a binary value classifying the input region as DMR or non-DMR. The loss function is binary cross-entropy, and the network optimizer is the Adam optimizer. To prevent overfitting the validation loss value is monitored; if it does not decrease after 5 epochs the training process is terminated.

Training a DL network requires hyper-parameter tuning. Several different parameters need to be tuned to improve the performance of a DL network model. Additional file 3: Table S1 shows the hyper-parameters for this network. As an example, one of the hyper-parameters to train a deep neural network is the depth of the network. Networks of different depths are trained to identify the network depth resulting in the best performance on a separate validation set. The depth of the networks varies from 1 to 5 blocks. Deeper networks can learn more complex classifiers, but risk overfitting the data. Shallower networks avoid overfitting by learning a more general classifier, but risk underfitting the data. Based on the accuracy and the precision scores, the best depth for the hybrid framework is 2.

After training the DL network, features are extracted from the first convolution layer. These features are used to re-express the data for input to the non-DL machine learning classifier. There are several options for using a DL network to construct features for a hybrid model. Autoencoder architectures can be used extract features in an unsupervised setting, but the non-DL classifier trained with autoencoder features performs worse than with features from a supervised network. The autoencoder features are used to regenerate the original input. However, classifier DL features re-express the data regarding the labels. Another option is to use recurrent neural networks, but training these networks is time-consuming compared to the CNN models.

### Machine learning classifier

DL networks need a large amount of data to train a strong classifier, compared to other ML methods. To overcome the need for large amounts of data, a hybrid learning method is used, where the DL network is used to learn new features that re-express the data for input to an ML classifier, which typically requires smaller amounts of data to achieve high accuracy. Another reason to use a non-DL machine learning classifier is to get a better understanding of why the classifier makes a particular prediction. For example, using a tree-based classifier provides the ability to rank the features and find the most important features.

One of the main issues with epigenetic datasets is that they are naturally imbalanced. Bagging and boosting are two commonly used methods in ML to address class imbalance. The bagging method uses multiple samples of the original dataset to learn an ensemble of different classifiers, which are collectively used to vote on the final classification. One of the best-performing bagging methods is Random Forest [75], which generates an ensemble of decision trees based on different random-selected subsets of the input features. Boosting also generates an ensemble of models, but in a sequential fashion, where each subsequent model is biased to focus on the errors of previous models. XGBoost [46] is one of the best methods for boosting, where the individual classifiers are decision trees. XGBoost uses gradient boosting where new models are created that predict the residuals or errors of prior models. Gradient boosting is a supervised learning method that classifies data by combining an ensemble set of estimators and weaker models. XGBoost is an efficient algorithm in terms of computation time and memory usage. Gradient boosting uses gradient descent in function space, which looks for nearby models that minimize the loss function (classification error). In constrast, XGBoost uses Newton Raphson in function space that considers models farther away that minimize classification error. The Newton–Raphson approach is accomplished by computing both the gradient and second-order gradient (hessian) of the loss function and using the ratio of the two as the error to minimize in the next classifier added to the boosting ensemble. Experimental comparisons between using Random Forest and XGBoost as the ML classifier in the hybrid approach resulted in XGBoost outperforming Random Forest in almost every case. So, the hybrid model uses XGBoost as the ML classifier. Like most tree-based classifiers, XGBoost can also output an importance ranking over the input features, which can be used to identify features in the DL network that are most important for making the final classification.

### Motif visualization

Feature motif visualization is accomplished by first representing the motif using the position weight matrix (PWM) method used in other work, including FactorNet [72], DanQ [48], and DeepBind [35]. The PWM is essentially the normalized distribution over the five possible bases (A,G,C,T,N) for each position in the DNA sequence. Visualizing the PWMs of a sequence helps to identify the parts of the sequence the neural network finds most relevant for predicting DMRs. The pysster package [52] is used to produce the PWMs and motif visualizations.

### Animal studies and breeding

Outbred Sprague Dawley SD male and female rats, (Envigo, Livermore, CA), were fed a standard diet with water ad lib and mated. Gestating female rats were exposed and offspring bred for three generations in the absence of exposure. The breeding strategy and details are described in the published literature cited. The F3 generation was aged to 1 year and pathologies assessed. Sperm were isolated and used for epigenetic analysis and correlated to individuals’ disease. Animals were sacrificed and disposed under WSU approved procedures. All experimental protocols for the procedures with rats were pre-approved by the Washington State University Animal Care and Use Committee (protocol IACUC # 2568), and all methods were performed in accordance with the relevant guidelines and regulations.

### Epigenetic analysis, statistics and bioinformatics

DNA was isolated from the purified sperm, as previously described [76]. Methylated DNA immunoprecipitation (MeDIP) followed by next generation sequencing (MeDIP-Seq) was performed. MeDIP-Seq sequencing libraries and next generation sequencing quality control were performed, as described in the cited studies. To ensure consistency across datasets, all DMR analyses were repeated using identical analysis parameters, including a 1000 bp genomic window size. As in the cited studies, the edgeR [77] p-value was used to identify differential sites. All molecular data has been deposited into the public database at NCBI under GEO #s: GSE113785 (vinclozolin), GSE114032 (DDT), GSE98683 (atrazine), GSE155922 (jet fuel), GSE157539 (dioxin), GSE158254 (pesticides), GSE158086 (methoxychlor), GSE163412 (plastics), and GSE152678 (glyphosate). R code computational tools are available at GitHub (https://github.com/skinnerlab/MeDIP-seq) and www.skinner.wsu.edu.

## Supplementary Information

**Additional file 1. Fig S1**: Deep learning DNA sequence features for non-DMRs. Sequence motif visualizations for the 11 non-DMR detector features out of the 32 features extracted from the DL model. Non-DMR detectors are those features whose average activation for non-DMR examples is greater than for DMR examples. The feature ID, motif visualization, and the difference between the average non-DMR activation and the average DMR activation are presented. A larger difference indicates a feature motif more biased toward non-DMRs

**Additional file 2. Fig S2**: The accuracy of different models (Hybrid, DL, DeepCpG, DeepSEA, and DanQ) for DMR prediction. Each line shows the accuracy of each approach when trained and tested on the DMRs and non-DMRs for an individual chromosome. The points at the far right for ‘All’ are the accuracies of these ‘all chr’ models on all the DMRs and non-DMRs across the entire genome.

**Additional file 3. Table S1**: Description of the hyper-parameters used to optimize the DL model. The underlined values resulted in the best-performing network

**Additional file 4. Table S2**: Summary of the datasets, including a brief description and the number of DMRs in the dataset due to the exposure. The number of non-DMRs is also shown, but they are determined from the genome, not individual exposure data

## Data Availability

All molecular data has been deposited into the public database at NCBI under GEO #s: GSE113785 (vinclozolin), GSE114032 (DDT), GSE98683 (atrazine), GSE155922 (jet fuel), GSE157539 (dioxin), GSE158254 (pesticides), GSE158086 (methoxychlor), GSE163412 (plastics), and GSE152678 (glyphosate). R code computational tools are available at GitHub (https://github.com/skinnerlab/MeDIP-seq) and www.skinner.wsu.edu. Availability at github.com/skinnerlab/DL-ML-Hybrid and github.com/holderlb/DL-ML-Hybrid.

## Abbreviations

DL: Deep learning
ML: Machine learning
DMRs: Differential DNA methylated regions
ncRNA: Non-coding RNA
DDT: Dichloro-diphenyl-trichloroethane
DEET: N,N-Diethyl-meta-toluamide
JP-8: Jet Fuel
MeDIP: Methylated DNA immunoprecipitation
