# Supplementary material for: Predicting environmentally responsive transgenerational differential DNA methylated regions (epimutations) in the genome using a hybrid deep-machine learning approach
Source: BMC Bioinformatics. 2021 Nov 30;22:575. doi: 10.1186/s12859-021-04491-z (PMC8630850; doi:10.1186/s12859-021-04491-z)
Supplement: Supplementary file 1 — Additional file 1. Fig S1: Deep learning DNA sequence features for non-DMRs. Sequence motif visualizations for the 11 non-DMR detector features out of the 32 features extracted from the DL model. Non-DMR detectors are those features whose average activation for non-DMR examples is greater than for DMR examples. The feature ID, motif visualization, and the difference between the average non-DMR activation and the average DMR activation are presented. A larger difference indicates a feature motif more biased toward non-DMRs [file 12859_2021_4491_MOESM1_ESM.pdf]

Supplemental Figure S1
Deep Learning DNA Sequence Features for Non-DMRs

ID	Motif Visualization	Average Difference
26	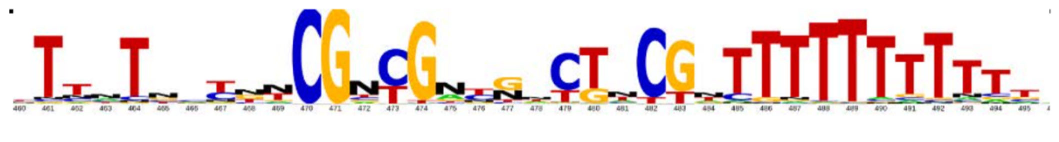	0.6013
13	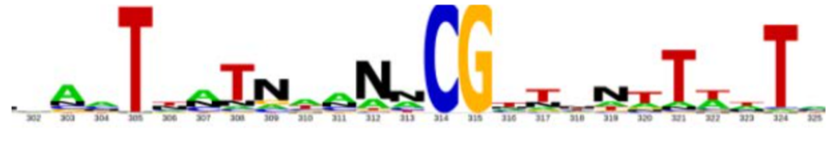	0.2895
8	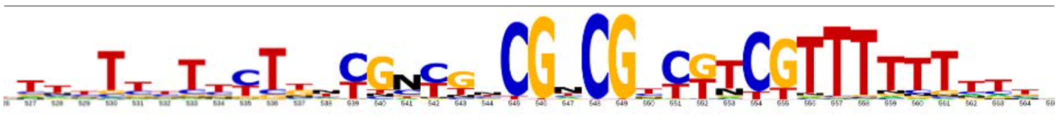	0.2845
31	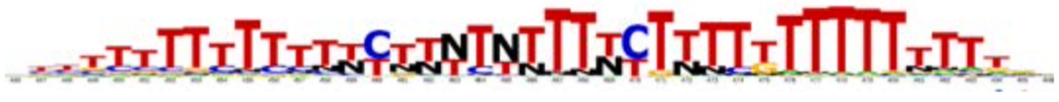	0.1888
20	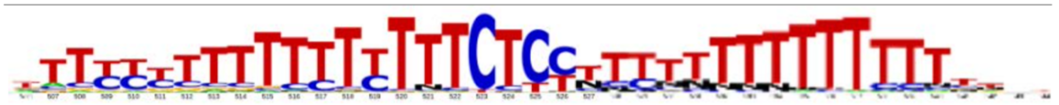	0.1879
30	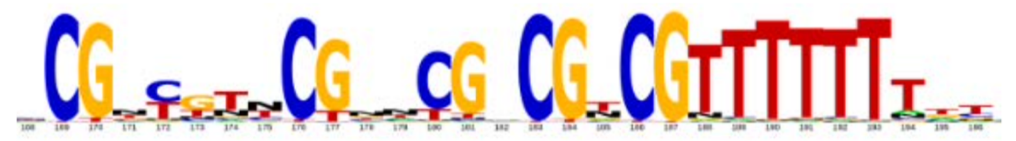	0.1886
27	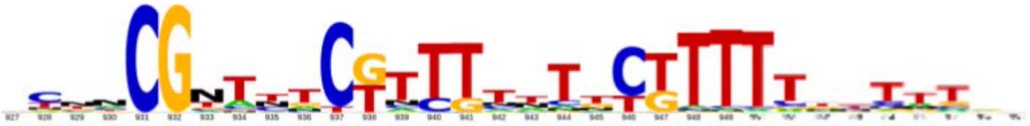	0.1692
22	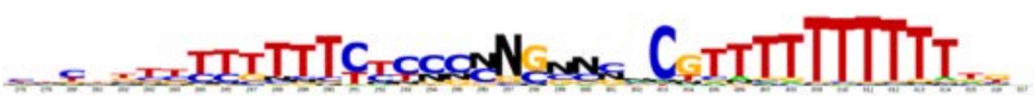	0.0988
23	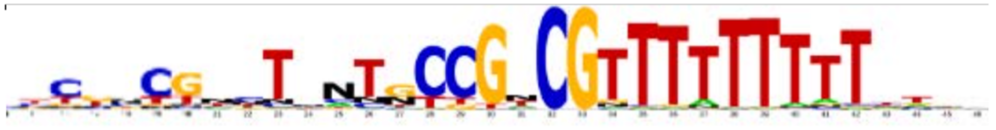	0.0852
11	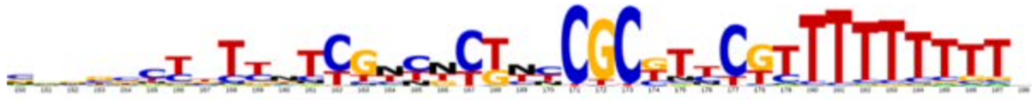	0.0476
7	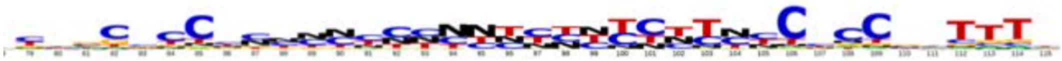	0.0235
