# Supplementary figures and images for: Predicting environmentally responsive transgenerational differential DNA methylated regions (epimutations) in the genome using a hybrid deep-machine learning approach
Source: BMC Bioinformatics. 2021 Nov 30;22:575. doi: 10.1186/s12859-021-04491-z (PMC8630850; doi:10.1186/s12859-021-04491-z)

Supplemental Figure S2

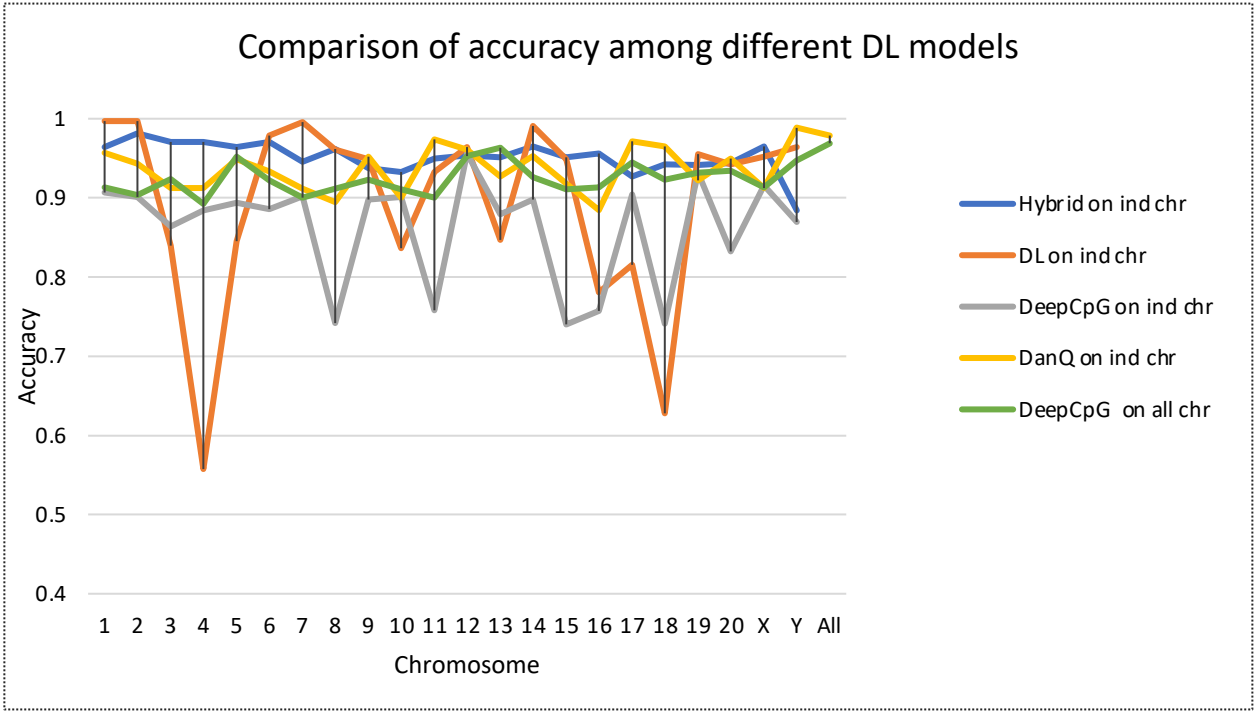

Supplement: Supplementary file 2 — Additional file 2. Fig S2: The accuracy of different models (Hybrid, DL, DeepCpG, DeepSEA, and DanQ) for DMR prediction. Each line shows the accuracy of each approach when trained and tested on the DMRs and non-DMRs for an individual chromosome. The points at the far right for ‘All’ are the accuracies of these ‘all chr’ models on all the DMRs and non-DMRs across the entire genome. [file 12859_2021_4491_MOESM2_ESM.pdf]
