# Supplementary material for: Predicting environmentally responsive transgenerational differential DNA methylated regions (epimutations) in the genome using a hybrid deep-machine learning approach
Source: BMC Bioinformatics. 2021 Nov 30;22:575. doi: 10.1186/s12859-021-04491-z (PMC8630850; doi:10.1186/s12859-021-04491-z)
Supplement: Supplementary file 3 — Additional file 3. Table S1: Description of the hyper-parameters used to optimize the DL model. The underlined values resulted in the best-performing network [file 12859_2021_4491_MOESM3_ESM.pdf]

Supplemental Table S1
Description of the hyper-parameters used to optimize the DL model. The underlined values resulted in the best-performing network.

Hyper-parameters	Values	Description
Number of blocks	2, 3, 4, 5	Depth of the network based on the number of blocks.
Epochs	50, 100, 200	Max number of epochs before training ends.
Patience	5, 10, 15, 20	Stopping conditions based on the number of epochs with no improvement in the validation loss.
Learning rate	0.001, 0.00001, 0.000001	Learning rate for Adam optimizer, decreased from the default value of 0.001 to smooth the learning curves.
Kernel size	10, 15, 20, 25, 30	Size of the convolution kernels.
Dense	128, 256, 512, 1024	Number of units in the dense layer.
Dropout	0.4, 0.5	Dropout rate.
