# Supplementary material for: Predicting environmentally responsive transgenerational differential DNA methylated regions (epimutations) in the genome using a hybrid deep-machine learning approach
Source: BMC Bioinformatics. 2021 Nov 30;22:575. doi: 10.1186/s12859-021-04491-z (PMC8630850; doi:10.1186/s12859-021-04491-z)
Supplement: Supplementary file 4 — Additional file 4. Table S2: Summary of the datasets, including a brief description and the number of DMRs in the dataset due to the exposure. The number of non-DMRs is also shown, but they are determined from the genome, not individual exposure data [file 12859_2021_4491_MOESM4_ESM.pdf]

Supplemental Table S2

Exposure	Description	#DMRs	#non-DMRs
DDT	Insecticide	121	126163
Atrazine	Herbicide	5	
Glyphosate	Herbicide	1	
Vinclozolin	Fungicide and pesticide	4	
Methoxychlor	Insecticide	63	
Pesticides	Includes the pesticides permethrin and N,N-Diethyl-meta-toluamide (DEET)	266	
Dioxin	Biproduct of the manufacture of chlorinated compounds, such as some herbicides, but also occurs naturally.	3169	
Plastics	Includes the plastics bisphenol A and phthalates	40029	
Jet Fuel	Hydrocarbon mixture used commonly by the military.	672	
